# Barriers and Facilitators of Health Literacy among D/deaf Individuals: A Review Article

**Published:** 2017-11

**Authors:** Tahereh NASERIBOORIABADI, Farahnaz SADOUGHI, Abbas SHEIKHTAHERI

**Affiliations:** 1. Dept. of Health Information Management, School of Health Management and Information Sciences, Iran University of Medical Sciences, Tehran, Iran; 2. Health Management and Economics Research Center, School of Health Management and Information Sciences, Iran University of Medical Sciences, Tehran, Iran

**Keywords:** Deafness, Health literacy, Barrier, Facilitator

## Abstract

**Background:**

The implication of health literacy is the ability of individuals to find, understand, and use their required health information from reliable sources. It is an indicator of the individuals’ participation in their own medical decision-making. Deaf individuals have limited health literacy and poor health status due to low literacy. Hence, this review was conducted to understand barriers and facilitators influencing health literacy among deaf community.

**Methods:**

We searched the ISI Web of Sciences, Scopus, and Medline from 1987 to 2016. Seventy-three papers were analyzed thematically.

**Results:**

We found three primary themes, including inadequate health literacy, barriers, and facilitators to accessing health information and health care services among deaf individuals. Facilitators were composed of four sub-theme including legal activities protecting the right of deaf patients to accessing health services, training health professionals about effective communication with deaf patients, providing sign language interpreter services, and developing deaf-tailored educational health programs and materials.

**Conclusion:**

Closing the deaf cultural gap and their limited access to health information are achievable through the removal of the communication barriers, allowing deaf individuals with more access to health learning opportunities, and informing the hearing community about the communicative skills of deaf individuals.

## Introduction

Health literacy (HL) is defined as ‘the knowledge, motivation, and a set of abilities required to access, understand, process, evaluate, and use health information to make judgments regarded to three-fold health domains as health care, disease prevention, and health promotion’ (1).

In fact, HL is the same general literacy affected by particular and general health-related factors (2). For example, the verbal and audio information exchanged through health care episodes is an active factor in patient-provider communication related to patient health outcomes. Therefore, healthcare providers (HCPs) have to use plain language and teaching back method in patient-HCPs communication (3).

Many HCPs are not often aware of inadequate literacy among patients causing them to overestimate the patients’ knowledge of medical conditions, disease prevention, and existing therapeutic options. Therefore, they may not make appropriate health educational advice during medical encounters (4, 5).

Little HL results in low participation in screening programs (6) and deprivation of receiving care efficiently among patients (7). Moreover, individuals with inadequate HL use preventive health services lower than health intelligent individuals (8). In addition, people with inadequate HL are unable to participate in self-care activities and comply with medical advice (9). Deafness, a prevalent problem around the world, remarkably influences the quality of life in hearing impaired individuals (10). Either many preventable diseases or conditions due to their limited HL threaten the health of the deaf community. The ignorance of HCPs on their health needs and lack of communication skills worsen their poor health status (11).

The aim of this narrative review was to consider both barriers and facilitators to HL for the deaf.

## Methods

### Search strategies

**Fig. 1:**
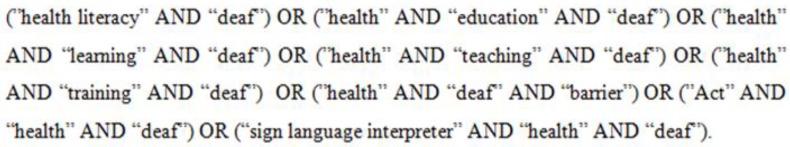
Keywords used for this study

We used the following search strategies in topic, title/abstract and keywords fields in the electronic databases of the ISI Web of Sciences, Scopus, and Medline (Fig. 1).

### Inclusion and exclusion criteria

Both English and non-English papers after translation into English were addressed in the review. Besides, that subject area was limited to articles or reviews. Therefore, short survey, brief research report, letter, book, book review, conference paper, and conference review were excluded.

### Analysis

Collectively, we retrieved 1173 papers from the ISI web of sciences (n=262), Scopus (n=677), and Medline (n=234). Screening the titles 1015 papers were eliminated due to duplication (n=402) and irrelevancy (n=613). Scanning the titles and abstracts additional papers were omitted due to the lack of accessibility to abstract (n=22), and full texts (n=63).

Included papers were reviewed by two researchers of this study individually and initial themes lists were created. Comparing these two themes lists similarities and differences were identified. In addition, to reduce disagreements, three researchers reviewed and revised the initial themes together. Creation of final themes and subthemes were the consensus-based process. Therefore, considering the aim of this study, we categorized papers into three primary themes and four sub-themes.

## Results

In total, 72 full texts published from 1987 to 2016 were included (12–73, 75–76, 78–81, 84, 86, 89, 90) and analyzed thematically and classified into following six tables.

Table 1 devotes to a summary of studies on inadequate HL among the deaf community. The findings focused on deaf people’s inadequate health knowledge. Table 2 relates to a summary of studies on difficulties in access to health information. Communication barrier is a primary challenge restricted deaf persons to access the health information and healthcare services (HCSs).

**Table 1:** A summary of studies on inadequate HL among the deaf community

***First Author; Reference Number***	***Methods***	***Findings***
Mallinson (12)	Pilot Study	Deaf more suffer from physical and mental consequences owing to the lack of deaf-friendly health services.
Oredugba (13)	Quantitative	Deaf children had poor knowledge and practice of oral dental health
Bat-Chava (14)	Qualitative	Deaf community has limited access to health information.
Orsi (15)	Quantitative	Low awareness of screening tests has been reported indicating uninformed decision-making in the deaf.
Goldstein (16)	Quantitative	American deaf high school students had limited awareness of HIV/AIDS.
Maddalena (17)	Qualitative	The deaf community had inadequate access to end-of-life care.
Wei (18)	Quantitative	Deaf students had poor knowledge and practice of oral health.
Berman (19)	Quantitative	Poor awareness and practice of breast cancer have been reported among deaf women due to unmet health information needs and communication barriers.
Napier (20)	Qualitative	The deaf community had limited access to preventive and ongoing health information due to limited English literacy.
McKee (21)	Quantitative	Low attainment of education increases the possibility of catching cardiovascular diseases among Deaf people (OR = 55.76). The lower the education level, the higher the likelihood of cardiovascular disease.
Sheppard (22)	Qualitative	Deaf community has unequal access to HCSs.
McKee (23)	Mixed Method	Researchers reported the high prevalence of low HL among the deaf is 6.9 times more than in hearing people. In addition, they stated that deaf individuals do not have access to mass media, health-care messages, and health-care communication among the deaf community due to communication and language barriers.
McKee (24)	Quantitative	Deaf use emergency services were more than in hearing persons (OR = 1.97), but the reasons are remained unknown.
Smith (25)	Qualitative	There is a high rate of catching cardiovascular disease among the deaf due to poor health knowledge.
Kushalnagar (26)	Quantitative	Low HL leads to difficulty in finding information in ASL accessible health website.
Kuenberg (27)	Review	There is a gap in health knowledge among deaf in the world.
Terry (28)	Mixed-method	Deaf individuals have limited access to sufficient and appropriate health care. They also have poor knowledge regarding health-related issues.

**Table 2:** A summary of studies on difficulties in access to health information

***Barrier***	***First Author; Reference Number***	***Methods***	***Findings***
**Legal**	Chilton (29)	Review	Deaf face difficulties in medical facilities due to either no adequate policies of providing SLISs or their reluctance to direct pay for this service.
	Steinberg (30)	Qualitative	Mistrust of HCPs and communication problems are the biggest challenges deaf patients face in mental health care.
**Interpersonal**	Kritzinger (31)	Qualitative	Deaf community has inadequate access to health information and services due to interpersonal factors, including independent thoughts, protectedness, and non-questioning attitudes.
	Witte (32)	Qualitative	Having difficulty in scheduling appointments and communication barriers have been identified as significant challenges.
	Chaveiro (33)	Qualitative	The communication barrier has led to social exclusion in the healthcare environment.
**Communication**	Bat-Chava (14)	Qualitative	Deaf individuals are unable to communicate with health providers.
	Folkins (34)	Quantitative	Deaf community has limited access to health information due to communication barriers.
	Groce (35)	Quantitative	The lack of effective communication with HCPs and access to health care facilities were reported.
	Scheier (36)	Review	Many HCPs do not understand how to improve communication with deaf patients.
**Language**	Mallinson (12)	Pilot Study	Deaf youth is at a higher risk of catching HIV due to the language barrier, stigma, and disparities faced in the health setting.
	Jones (37)	Mixed Method	Deaf individuals face oral and printed language barriers.
	Kritzinger (31)	Qualitative	Deaf individuals have limited access to health-care services due to communication barriers and interpersonal factors.

**Table 3:** A summary of studies on legal activities to improve HL among deaf community

***First Author; Reference Number***	***Methods***	***Findings***
Chacko (38)	Review	Under the Section 504 of the U.S. Rehabilitation Act, effective deaf patient-HCPs communication is mandatory during emergency health service delivery.
Chilton (29)	Review	The Section 504 of the Rehabilitation Act (Public Law 93-112) and Title III of the Americans with Disabilities Act (ADA) are facilitators in this field.
Ubido (39)	Quantitative	HCPs’ ignorance of the Disability Discrimination Act leads to legal action against them.
Chaveiro (40)	Review	The importance of using sign language in a health setting to overcome the communication barrier and improve health outcomes for the deaf community has been recognized under Federal Law 10.436/02.
Pereira (41)	Qualitative	Under the decree, 5626/05 under Federal Law 16.436/02, HCPs have to be familiar with sign language or hiring sign language interpreters.
Chan (42)	Review	Providing SLISs is mandated under the ADA.
Haricharan (43)	Quantitative	Under the Convention on Rights of Persons with Disabilities, providing qualified sign interpreter services is mandatory, as a constitutional right of access to health care is a pre-requisite of information accessibility in South Africa.
Brown (44)	Review	Health care entities have to be obliged to facilitate deaf patients’ accessibility to health services under the ADA through hiring a qualified interpreter and through needed communication technologies.

Table 4 summarizes studies on sign language interpreting services (SLISs) in health care. It shows that qualified SLIs mediated deaf patient-HCPs communication. Table 5 relates to studies on training HCPs about deaf culture and developing communication proficiency. Additionally, health professionals would provide deaf-tailored health services if they have attended in training courses on developing and improving communication skills.

**Table 4:** A summary of studies on sign language interpreting services in health care

***First Author; Reference Number***	***Methods***	***Findings***
MacKinney (45)	Quantitative	The presence of full-time interpreters in medical encounters leads to a higher level of satisfaction with physician communication and better preventive health outcomes.
Chilton (29)	Review	The effective way of communication in response to the deaf patient is using a SLI rather than paper and pen, or lip-reading.
Cardoso (46)	Qualitative	The presence of qualified SLI is necessary for having effective patient-HCPs communication.
McKee (47)	Quantitative	SLIs may reduce potential risks of miscommunication between deaf patients and HCPs.
Major (48)	Qualitative	The health lexicon of Auslan is underdeveloped; therefore, professional interpreters have to play a mediating role in health terms and communication to improve health terminology comprehension among deaf patients.

**Table 5:** A summary of studies on training HCPs about deaf culture and developing communication proficiency

***First Author; Reference Number***	***Methods***	***Findings***
Margellos-Anast (49)	Quantitative	Training HCPs about deaf culture and communication is a need.
Mathews (50)	Quantitative	First-year pharmacy students confirmed the effectiveness of role-playing exercises on patient-HCPs communication.
Hoang (51)	Quantitative	Training medical students in deaf cultural competency may lead to improving health outcomes for the deaf community.
Thew (52)	Quantitative	Medical students in the clinical-oriented stage confirmed the long impact of the “Deaf Strong Hospital Program a year after launching the program.
Nagakura (53)	Quantitative	Students are not familiar with deaf culture due to the limited related academic training.
Adib-Hajbagheri (54)	Quantitative	Nursing students had poor knowledge and practice of interaction with deaf patients.
Lapinski (55)	Quantitative	Confidence and knowledge of the way of communication with the deaf have been increased over the course of “deaf culture and primary medical ASL”.
Velonaki (56)	Quantitative	Educating nurses for developing their proficiency in communication with the deaf was welcomed.
Ferguson (57)	Quantitative	Pharmacists have to educate about deaf culture to improve their communication skills.
Yuksel (58)	Qualitative	Simulated patient method improved the communication skills of nursing students in caring for deaf patients.

Table 6 shows a summary of studies on developing deaf-tailored materials and programs. Notably, it focused on the effectiveness of holding deaf-tailored educational health programs.

**Table 6:** A summary of studies on developing deaf-tailored materials and programs

***First Author; Reference Number***	***Methods***	***Findings***
Gregg (59)	Qualitative	Developing a health educational program requires addressing little proficiency in spoken and written languages, need for the presence of SLI, and limited readability skills among deaf.
Bat-Chava (14)	Qualitative	Developing and disseminating deaf-tailored educational materials and providing further deaf medical services were recommended.
Margellos-Anast (49)	Quantitative	Deaf tailored medical education materials have to be developed because of a lower level of cardiovascular knowledge for deaf people comparing with hearing people.
Kaskowitz (60)	Quantitative	The deaf community health knowledge was increased after viewing a cancer educational video in ASL.
Jones (61)	Quantitative	The self-efficacy of deaf adults for health behaviors was increased after the completion of heart health intervention.
Choe (62)	Quantitative	Deaf women’s knowledge of cervical cancer was increased after one viewing of a related graphically enriched educational video in ASL.
Wilson (63)	Quantitative	The efficiency and cost-effectiveness of telehealth services to the deaf community, and their satisfaction with these services was demonstrated.
Jones (64)	Quantitative	Deaf community appreciated an interactive web-based education.
Berman (65)	Quantitative	Tobacco-related knowledge, attitude and practice were affected by the deaf-tailored tobacco-use prevention curriculum so that tobacco exposure was decreased and an anti-tobacco attitude increased.
Sadler (66)	Quantitative	Knowledge of cancer was increased after viewing the deaf-tailored videos among the deaf.
Hickey (67)	Quantitative	The knowledge of breast cancer in deaf women was increased after viewing the educational video.
Chiriac (68)	Quantitative	Translation of medical knowledge and concepts into the sign language through the avatar interface is necessary in developing e-health systems for deaf users.
Ahmadi (69)	Mixed Method	The health education software facilitates efficient learning of child health topics for teachers and parents of deaf students.

## Discussion

### (a) Inadequate HL for the deaf

Deaf individuals are unaware of the health-disease process and ignorant of health knowledge due to poor access to deaf-tailored health information sources, and limited access to mass media and healthcare messages owing to communication and language barriers (20, 26, 59, 60). Hence, they have limited knowledge of medical conditions and symptoms of diseases (32, 52, 61, 62), inadequate awareness of disease prevention (71, 72) medical screening tests (15), and preventive health services (47). Hence, it is necessary to identify the barriers to accessing health information for the deaf.

### (b) Barriers to access health information and HCSs among the deaf

According to Helen Killer’s comment about hearing loss, communication is vital for deaf individuals (74). Nevertheless, communication and language barriers have been recognized as the indubitable underlying causes of the gap in health knowledge among the deaf community (27, 75).

Communication barriers, lack of education, and limited access to deaf-tailored health information are significant contributors to poor health among the deaf (76). The lack of HCPs in understanding the deaf culture and required communication skills to interact better with deaf individuals is the leading cause of communication problem deaf individual face in health settings (77). Moreover, the lack of knowing how to communicate with hearing HCPs is more bothersome than hearing loss, particularly when the preferred communication method is ASL (78). Communication and language barriers may lead to negative consequences for deaf individuals, including isolation, low self-esteem, abuse, and inadequate health care (79). In addition, deaf person’s interpersonal factors (31), and the ignorance of HCPs on the rights of deaf individual’s access to HCSs make the accessibility of HCSs for deaf individuals, a serious issue (29).

### (c) Facilitators in access to health information and HCSs for the deaf

To remove the obstacles to improving HL “facilitators” composed to the following four sub-themes including protecting deaf people right to access to health services, providing sign language interpreter, training health providers about deaf culture, and developing deaf-tailored programs in health education.

### (c_1_) Legal activities protecting deaf individual’s right to access HCSs

Deaf individuals caught in a dangerous situation regarding the source of HL process. As for enhancing HL, deaf persons have the right to have equal access to HCSs under different regulations in which having effective communication with deaf individuals and providing SLISs are recognized in particular in providing emergency health care (38, 81). Health care entities obligated to provide communication technologies and SLIs for deaf individuals (76), and disobedience of health care entities to provide a sign language interpreter can incur heavy penalties and litigation (39).

### (c_2_) Providing sign language interpreting services

Given the possibility of failed lip reading attempts in health-related situations and the patients’ concerns about their confidentiality violations during medical encounters and procedures in the presence of family members, sign language interpreter service is compulsory (76). However, the ignorance of physicians to their liabilities to provide interpreters for their deaf patients has been demonstrated in other previous studies (82, 83).

In addition, the association between the presence of a full-time interpreter in medical encounters and more satisfaction with physician communication, and improved preventive health outcomes have been confirmed in another study (45).

Developing proficiency in mutual apprehension, communication, and negotiation about common professional tasks among HCPs and interpreters to provide a flourishing bilingual medical encounter is a requirement (84).

### (c_3_) Training HCPs about deaf culture

Holding academic training courses on effective communication skills are required for closing the miscommunication between deaf individuals and HCPs (54). These educational interventions have influenced on gaining practical experience regarding deaf culture and required communication skills in communication with deaf individuals. Since the possibility of interacting with deaf individuals may occur, HCPs have to develop proficiency in managing HCSs to deaf individuals (85, 86).

In this line, the practical courses are more efficient than traditional teaching methods (52). The effectiveness of the interventions has been confirmed through a training workshop with osteopathic students (55). Educating nurses for developing their proficiency in communication with the deaf was welcomed (56).

### (c_4_) Developing deaf-tailored programs in health education

In fact, deaf individuals have inconsistent health information about conditions such as cardiovascular risk factors (87), and medical screening tests (15). Therefore, to decrease the health inequalities to access preventive health services, educating deaf community is of particular significant (88). Many health education interventions have conducted through different platforms ranged from booklets, slides, videos to interactive web-sites over the last few years. These interventions may result in better comprehension of health-related issues using native sign language, open captioning, and images and videos (89, 90).

### Implication for promotion of HL among deaf community

The deaf community might have better access to reliable health information sources through educating sign language-fluent HCPs and training sign language interpreters proficient in basic medical concepts.

## Conclusion

To date, many barriers have been identified by which deaf individuals have no equal access to health information and healthcare services. These barriers are classified into five groups, including interpersonal factors, cultural, language, and communication barriers.

Deaf community has common characteristics like inadequate HL, poor health, and social exclusion in health settings. In fact, deaf individuals are passive in their personal health decision-making due to their particular conditions they experience. As a result, they cannot overcome these barriers by themselves.

Meeting deaf individuals health needs, and promoting their health status involve increasing incidental health learning situations, providing sign language interpreter services, developing deaf-tailored educational programs, and training HCPs about deaf individuals’ health needs.

## Ethical Considerations

Ethical issues (Including plagiarism, informed consent, misconduct, data fabrication and/or falsification, double publication and/or submission, redundancy, etc.) have been completely observed by the authors.
